# The Role of Hemogram-Derived Inflammation Indices for the Prediction of Nausea and Vomiting in Pregnancy and the Need for Hospitalization

**DOI:** 10.3390/diagnostics16050669

**Published:** 2026-02-26

**Authors:** Belgin Savran Üçok, Murat Levent Dereli, Sadun Sucu, Sadullah Özkan, Dilara Kurt, Ahmet Kurt, Fahri Burçin Fıratlıgil, Kadriye Yakut Yücel, Şevki Çelen, Ali Turhan Çağlar

**Affiliations:** 1 Ankara Etlik City Hospital, Ankara 06170, Turkey; dr.belgin@gmail.com (B.S.Ü.); medical.academic.sucu@gmail.com (S.S.); sadullahozkan@gmail.com (S.Ö.); dilarasarikaya30@gmail.com (D.K.); mflkurt@gmail.com (A.K.); md.fahri@gmail.com (F.B.F.); yakutkadriye@hotmail.com (K.Y.Y.); sevkicelen@yahoo.com (Ş.Ç.); turhan_caglar@yahoo.com (A.T.Ç.); 2 Denizli State Hospital, 20010 Merkezefendi, Turkey

**Keywords:** complete blood count, hemogram, hyperemesis gravidarum, index, pan-immune value, severity

## Abstract

**Objective:** To investigate the association between inflammatory indices derived from complete blood count, including the systemic immune-inflammation index (SII), systemic inflammatory response index (SIRI), and pan-immune inflammation value (PIV), in predicting nausea and vomiting in pregnancy (NVP). **Methods:** Women diagnosed and treated for NVP at a tertiary care hospital between 2016 and 2021 were retrospectively analyzed. A total of 278 eligible patients with NVP and 278 gestational age-matched healthy pregnant women were included. Patients with NVP were categorized as having mild (*n* = 58), moderate (*n* = 140), or severe NVP (*n* = 80). Patients with moderate and severe NVP, who almost always required hospitalization, were grouped together and assigned to the inpatient treatment group. The groups were then compared. **Results:** SII and PIV were significantly higher in the NVP group than in the control group (*p* < 0.001 for both). In addition to SIRI, SII and PIV were also significantly higher in both the moderate NVP and HG groups compared to the mild NVP group (*p* = 0.017, 0.040, and 0.038, respectively, and *p* = 0.003, 0.009, and 0.006, respectively). SII, with a cut-off value of >966 × 10^3^/μL (63.67% sensitivity, 68.35% specificity), showed the best discriminatory performance for predicting NVP (*p* < 0.001), but there was no significant difference among SII, SIRI, and PIV in predicting the need for hospitalization. **Conclusions:** Our results show that there may be an association between high SII and PIV and an increased risk of developing NVP. In the future, after sufficient research, among these complete blood count-based inflammatory indices, SII may become an important component of regression models used as a screening tool to predict NVP, particularly in cases requiring inpatient care.

## 1. Introduction

Morning sickness, also known as mild nausea and vomiting in pregnancy (NVP), is a common symptom of pregnancy, especially in the first trimester. It is usually at its worst in the morning, but can occur at any other time of day [1]. Hyperemesis gravidarum (HG), on the other hand, is a condition associated with severe NVP and affects 0.3–10.8% of all pregnancies [1,2]. This pregnancy-specific condition can lead to serious problems, such as dehydration, electrolyte imbalance, malnutrition, weight loss and psychological disturbances in the mother, as well as growth retardation, neurological and as yet unknown effects on the developing offspring [3]. As HG may be associated with serious consequences, prediction, early detection and appropriate treatment planning of women at high risk of HG is crucial.

Although there are several theories that suggest endocrinological factors (human chorionic gonadotropin, thyroid hormones, estrogen and other placental hormones), genetic predisposition and psychological susceptibility, the exact physiopathological process has not yet been clarified [3,4]. During embryo implantation and trophoblastic invasion, a local proinflammatory response is triggered at the implantation site, which plays a crucial role in maintaining further embryo invasion, differentiation and placental development [5,6]. Any abnormality in this process can manifest as altered inflammation. Several inflammation biomarkers, such as tumor necrosis factor alfa, interleucin-6, neopterin and vaspin, have been studied and found to be partially involved in the etiology of HG [7,8]. Since these biomarkers cannot be used universally due to the high cost and difficulty of integrating them into clinical practice, inflammation markers derived from a simple complete blood count (CBC) appear to be more attractive for predicting diseases and/or their prognosis where altered inflammation plays an important role in pathogenesis. Accordingly, the relationship between CBC-derived inflammation parameters, including neutrophil-to-lymphocyte ratio (NLR), platelet-to-lymphocyte ratio (PLR) and monocyte-to-lymphocyte ratio (MLR) and the severity of HG has already been investigated [9]. Therefore, the hypothesis of the current study was based on altered inflammation, which is considered an important factor in the development of NVP and HG.

Research in this area continues to search for more relevant and useful predictive tools at a reasonable cost for predicting NVP and its severity. In this context, we aimed to investigate the role of systemic immune-inflammation index (SII), systemic inflammatory response index (SIRI) and pan-immune inflammation value (PIV) in the first trimester in predicting women at high risk of developing moderate NVP and HG.

## 2. Materials and Methods

### 2.1. Study Design

This study was conducted as a retrospective case-control study of 278 women with singleton pregnancies diagnosed with NVP at a large tertiary care hospital between 1 January 2016 and 31 December 2021. We included 278 gestational age-matched healthy women with singleton pregnancies as controls. The ethics committee approved the conduct, protocol and procedures of the study (22 February 2022, 03/26). All participants have been given informed consent to participate in the research. After approval, a retrospective review of the patients’ medical records was carried out.

### 2.2. Definitions, Characteristics of Study Population, Patient Selection

Pregnancy up to the 14th week of gestation was referred to as the first trimester. Gestational age was calculated from the first day of the last menstrual period and confirmed by sonographic measurement of the crown-rump length (CRL). If the calculation contained fractions of days, the gestational age was rounded up or down to the nearest whole week. The modified pregnancy-unique quantification of emesis and nausea (PUQE) index, which consists of the assessment of three components, including the duration of nausea in hours, the number of vomiting episodes, and retching within a day, was used to assess the severity of NVP [10]. In our study, the severity of NVP was categorized using the PUQE score: mild (4–6), moderate (7–12) and severe (≥13). Patients with moderate and severe NVP, who almost always require inpatient treatment, were studied together under the name ‘inpatient treatment group’.

The exclusion criteria were divided into two main categories, including situations associated with nausea and vomiting (gastroenteritis, cholecystitis, hepatitis and other diseases of the gastrointestinal tract, diabetic ketoacidosis, thyroid disease and neurological diseases, that can cause increased intracranial pressure and induce vomiting) and inflammatory diseases (pelvic inflammatory disease, coronavirus infections and other acute infections, autoimmune diseases, liver and/or kidney failure, diabetes mellitus, cardiovascular diseases). Women with altered inflammatory status or altered hemogram results due to anti-inflammatory medication and/or corticosteroids at the time of blood sampling, multiple pregnancies, molar pregnancies and women without a hemogram collected ≤5 weeks’ gestation or with missing data were also not included in the study.

**Figure 1:**
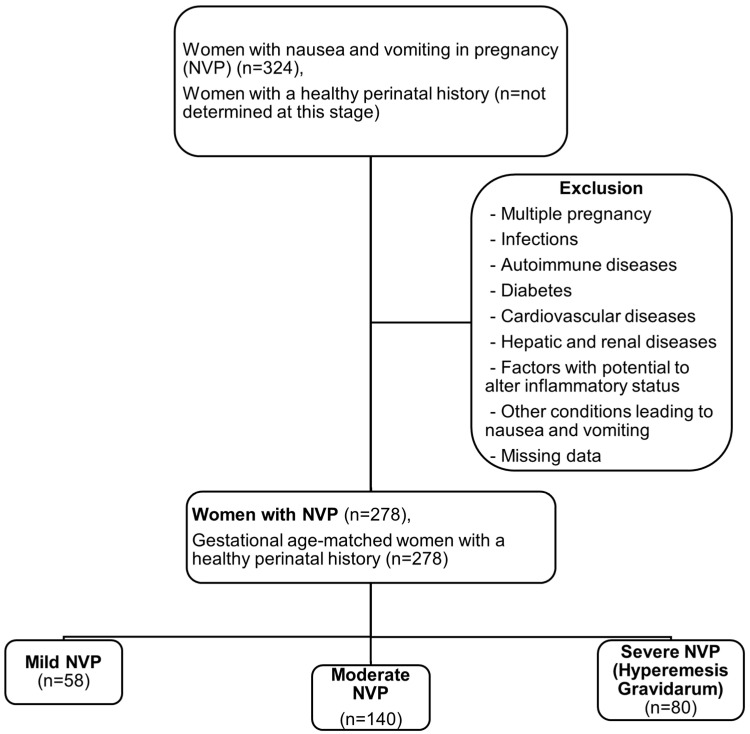
Flowchart for dividing the NVP group.

After applying the exclusion criteria, randomization for the control group was performed by including all gestational-age-matched healthy pregnant women who were admitted to the outpatient clinic in chronological order immediately following each woman’s admission to the NVP group. A total of 324 women were diagnosed with NVP during the study period. After exclusion, 278 eligible participants with NVP (NVP group) and 278 gestational age-matched healthy pregnant women (control group) were included in the study. The NVP group was divided into three subgroups according to severity: mild NVP (*n* = 58, 20.9%), moderate NVP (*n* = 140, 50.3%) and severe NVP (*n* = 80, 28.8%) (Figure 1).

### 2.3. Imaging Methods, Laboratory Measurements and Study Variables

Obstetric sonographic examinations were performed with a GE Voluson 730 Expert ultrasound machine (General Electric Medical Systems, Milwaukee, WI, USA). On admission to the clinic, blood samples for hemogram were collected in tubes containing ethylenediaminetetraacetic acid and analyzed within 30 min of receipt using a Mindray BC-6800 hematology analyzer (Mindray Medical International Limited, Shenzhen, China). Blood samples for analysis of biochemical parameters were placed in serum separator tubes containing gel and analyzed within 30 min of receipt using a Roche Cobas e801 chemiluminescence immunoassay analyzer (Roche Diagnostics International Limited, Rotkreuz, Switzerland).

Hemogram-derived inflammation indices, including SII, SIRI, and PIV, were calculated from retrospectively reviewed reports of hemograms obtained at the first prenatal examination at or before the fifth week of gestation, provided that participants had no signs of nausea or vomiting at that time. The associated formulas for inflammation indices were as follows: “SII = neutrophil count (μL) × platelet count (μL)/lymphocyte count (μL); SIRI = neutrophil count (μL) × monocyte count (μL)/lymphocyte count (μL); PIV = neutrophil count (μL) × platelet count (μL) × monocyte count (μL)/lymphocyte count (μL) [11].”

First-trimester clinical characteristics, including age, body-mass index (BMI), comorbidities, obstetric history, laboratory findings, hemogram parameters, and CRL measurements, were obtained from the hospital database.

### 2.4. Statistical Analysis

All statistical analyses were performed using the R Statistical Software version 2021.09.4+403.pro3 (R Foundation for Statistical Computing, Vienna, Austria). Shapiro–Wilk tests were used to determine normality. Descriptive analyses for the non-normally distributed numerical data were performed using medians and quartiles (quartile 1–quartile 3). Kruskal–Wallis and Mann–Whitney U tests were performed to compare these parameters between groups. Bonferroni correction was used to adjust for multiple comparisons. Descriptive analyses for the categorical variables were performed using frequencies and percentages. The relationships between categorical variables were analyzed with the chi-square test or Fisher’s exact test. The predictive power of various parameters that can be used to discriminate patients requiring inpatient treatment (moderate NVP and HG) was analyzed using receiver operating characteristics (ROCs) curve analysis. When a significant cut-off value was determined, sensitivity, specificity, area under the curve (AUC), positive likelihood ratio, and negative likelihood ratio were reported. The ROC curves and AUC values of these parameters were then compared with each other. For the multivariate analysis, the factors identified in the univariate analyses were included in the binary logistic regression to identify additional independent predictors of NVP. A *p*-value of less than 0.05 was considered a statistically significant result.

## 3. Results

A comparison of the baseline characteristics and clinical variables of the participants in the NVP and control groups was presented in Table 1. Maternal age and gestational age were not different, while BMI, gravidity and parity were significantly higher in the control group (*p* = 0.001 for all). Of the hemogram-derived inflammation indices, SII and PIV were significantly higher in the NVP group than in the control group [1173 (771–1702) vs. 774 (599–1089), *p* < 0.001; and 487.1 (321.7–816.8) vs. 397.7 (283.9–663.4), *p* < 0.001], while SIRI did not differ between groups.

Further comparison of baseline characteristics and clinical variables between the three subgroups of NVP classified by severity revealed no significant differences (Table 2). On the other hand, SII, SIRI and PIV were significantly higher in both the moderate NVP and HG groups than in the mild NVP group [(*p* = 0.017, 0.040 and 0.038, respectively) and (*p* = 0.003, 0.009 and 0.006, respectively)], while there were no significant differences between the moderate NVP and HG groups.

According to the ROC curve analysis performed to investigate the discriminatory power of SII and PIV to predict pregnancy in which NVP will develop, the AUC values were 0.708 and 0.593, respectively. The cut-off values for SII and PIV were >966 × 10^3^/μL (63.67% sensitivity, 68.35% specificity) and >348 × 10^6^/μL^2^ (73.02% sensitivity, 41.37% specificity), respectively. In addition, ROC curve analysis to examine the discriminatory power of SII, SIRI, and PIV to predict pregnancy with NVP requiring hospitalization yielded AUC values of 0.639, 0.625 and 0.627, respectively. The cut-off values for SII, SIRI and PIV were >1056 × 10^3^/μL (64.55% sensitivity, 68.97% specificity), 32.12 × 10^3^/μL^2^ (77.27% sensitivity, 43.10% specificity) and >350 × 10^6^/μL (77.27% sensitivity, 46.55% specificity), respectively (Table 3).

**Figure 2:**
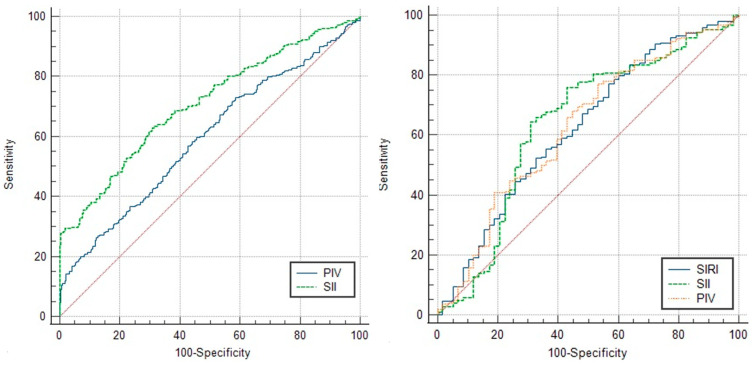
Comparison of the receiver operating characteristic (ROC) curves of SII, SIRI and PIV for the prediction of NVP and inpatient treatment need.

Comparisons of the ROC curves of these indices for predicting NVP and inpatient treatment needs are shown in Figure 2.

Comparison of overall performance in predicting NVP development showed that SII was better than PIV (*p* < 0.001), while there was no significant superiority between SII, SIRI and PIV in predicting inpatient treatment need (Table 4).

In the logistic regression analysis evaluating predictors of NVP, both SII and PIV were significant risk factors. In the unadjusted model, SII was significantly associated with increased risk of NVP (OR: 1.187, 95% CI: 1.138–1.239, *p* < 0.001). This association remained significant after adjustment for BMI and gravidity (OR: 1.163, 95% CI: 1.114–1.215, *p* < 0.001) and after adjustment for BMI and parity (OR: 1.163, 95% CI: 1.115–1.214, *p* < 0.001). Similarly, PIV was significantly associated with NVP risk in the unadjusted model (OR: 1.103, 95% CI: 1.052–1.157, *p* < 0.001). This relationship persisted after adjustment for BMI and gravidity (OR: 1.085, 95% CI: 1.034–1.139, *p* = 0.001) and after adjustment for BMI and parity (OR: 1.086, 95% CI: 1.034–1.140, *p* = 0.001). These findings indicate that the predictive value of SII and PIV for NVP is independent of BMI, gravidity, and parity. (Table 5).

## 4. Discussion

While mild NVP is a common pregnancy condition, especially in the first trimester, the etiopathologic mechanism leading to severe NVP, also known as HG, is not yet fully understood, and multiple factors are thought to play a role in the etiology [12]. The risk of HG is increased by known risk factors such as multiple pregnancies, molar pregnancies and pre-pregnancy risk factors, such as underweight, primiparity, hyperthyroidism, asthma, motion sickness and gastrointestinal disorders [13]. However, current knowledge of other potential mechanisms involved in pathogenesis is limited, as the studies investigating HG generally have small sample sizes, leading to inconclusive results. In this regard, HG is well defined by a recently published study with a large sample size over a long period of time, which emphasized that pregnancies with HG differ not only from pregnancies of women who have never experienced HG, but also from other non-hyperemetic pregnancies of women with HG [14].

Prediction and early diagnosis of HG is crucial to prevent serious maternal, fetal and neonatal consequences, such as vitamin B1 and K deficiencies, Wernicke’s encephalopathy, dehydration, electrolyte imbalance, malnutrition, preterm birth, fetal growth restriction and as yet unknown effects on the offspring [15,16,17,18,19]. This is especially important for pregnant women in rural areas who live far from health centers and lack the means for frequent visits, as well as for women who do not seek medical care due to cultural or financial constraints [20].

Although several studies have investigated biomarkers such as leptin, nesfatin-1, ghrelin, obestatin, and prealbumin, their role in predicting HG remains unclear due to inconsistent results and small sample sizes [12,21,22,23]. To date, there are no generally accepted clinical tools that can accurately predict the development of HG. Different studies have reported contradictory results in women with HG; some found higher, while others found lower leptin levels in women with HG compared to controls [21,23]. Similarly, nesfatin-1 levels were higher in women with HG compared to controls in the study by Gungor et al., while Ozturk et al. found no difference [22,23]. The sample sizes in all these studies were insufficient, and BMI-adjusted values could not be compared because no data on patients’ BMI measurements were available. In addition to these biomarkers, ultrasound findings have also been examined for HG prediction. A study by Yıldız et al. found that placental thickness and free beta-hCG levels were higher in patients with HG [24]. It is already known that a high beta-hCG level is associated with HG. Since the amount of placental mass correlates with the beta-hCG level, placental size is indirectly related to HG. However, accurate measurement of placental thickness may not be possible due to many factors, including the thickness of subcutaneous adipose tissue, placental localization, intra- and inter-observer variability, and the difficulty of standardizing the part to be measured.

As inflammation indices derived from a single hemogram have gained attention in recent years and represent an inexpensive, widely available, easily applicable, and interpretable tool with the potential to predict the development, prognosis, and severity of diseases with altered inflammation, such as HG, we conducted this study. Furthermore, to our knowledge, this article is the first to investigate the association between SIRI, PIV, and NVP, as well as their severity.

The main findings of this study were as follows: 1. SII and PIV were significantly higher in the NVP group than in the control group. An SII with a cut-off value of >966 × 10^3^/μL had greater discriminatory power in predicting NVP than a PIV with a cut-off value of >348 × 10^6^/μL^2^. 2. SII, SIRI, and PIV were significantly higher in both the moderate and severe NVP groups than in the mild NVP group, while there were no significant differences between the moderate NVP and HG groups. However, distinguishing between those who develop moderate or severe NVP (aka HG) does not contribute to clinical practice, as patients with both types almost always require inpatient treatment. On the other hand, SII, SIRI, and PIV can be used in models to differentiate between patients who will develop mild NVP and those who will develop moderate and/or severe NVP, as their discriminatory power was the same.

Inflammation indices and cell lines derived from the hemogram have previously been studied in relation to NVP. In the study by Tayfur et al., neutrophil, lymphocyte, and platelet counts, as well as NLR, PLR, and plateletcrit, differed significantly between the HG and control groups [25]. However, because altered inflammation is a complex process, it is neither realistic nor meaningful to make decisions based on a single cell line, such as neutrophils, lymphocytes, or platelets alone. For example, some conditions in the study population can significantly affect a single inflammatory cell line (e.g., primary thrombocytosis, parasitic diseases, certain viral diseases). Therefore, investigating the relationship between inflammation and the disease by considering only one inflammatory cell line—especially if it is the affected line—may lead to erroneous results. Instead, using inflammatory indices that evaluate multiple inflammatory cell lines together will result in a lower error rate in such situations. In this context, we investigated SII, SIRI, and PIV, which together assess different inflammatory cell lineages and have previously been studied for the prediction and prognosis of many diseases in which inflammation plays a role [26,27,28,29,30]. In a recent cohort study by Beser et al., the clinical utility of SII in predicting the severity of HG was considered limited due to its relatively low sensitivity and specificity (both 59%) [28]. In contrast, we evaluated the predictive utility not only of SII but also of other hemogram-based inflammation indices, PIV and SIRI, in NVP. SII and PIV were significant in predicting NVP, with sensitivity and specificity of 63.67% vs. 73.02% and 68.97% vs. 43.10%, respectively. SII was superior to PIV in predicting NVP. Additionally, we defined the inpatient treatment group as women with a PUQE score ≥ 7 (moderate or severe NVP) who almost always required hospitalization, and examined the same parameters to predict which women would require inpatient treatment. All three indices, which were not superior to each other, were significant in predicting the need for hospitalization. Furthermore, in the study by Tayfur et al., all women with NVP of varying severity were grouped together under the term “HG,” which should only refer to patients with severe NVP as defined in the same study [25]. In addition, the range of gestational age at the time of hemogram sample collection in that study was quite wide (before 22 weeks’ gestation) [25]. Since hemogram parameters vary with increasing gestational age, including pregnant women with a wide range of gestational ages leads to less consistent results.

Consistent with the literature, our study found that patients without NVP had higher BMI, gravidity, and parity than those with NVP. A higher BMI is associated with a lower rate of NVP, and since previous NVP is a significant risk factor for recurrence, it is not surprising that women who have experienced NVP and HG are less likely to want to become pregnant again. The main limitation of this study is its retrospective design, as recording bias may affect data accuracy and there is a risk of information and selection bias. Additionally, the generalizability of the proposed cut-off values may be limited by the single-center setting. Although the sample size is appropriate for a case-control study, it is relatively small, particularly for subgroup analyses or strong verification of several cut-off values, and therefore lacks statistical power. To reduce temporal bias, we selected the next healthy pregnant woman after the outpatient clinic admission of an NVP case, which introduced the potential for selection bias. Although calculating these inflammation indices may seem complicated, the results can be easily integrated into hemogram reports using software. The major strength of this study is that it was conducted in a large tertiary reference hospital using consistent algorithms for diagnosis, treatment, and follow-up.

## 5. Conclusions

Our results suggest an association between high SII and PIV levels and an increased risk of NVP. Furthermore, SII, SIRI, and PIV may be important components of future regression models designed to identify patients who will develop moderate or severe NVP to facilitate treatment planning, as these patients are at high risk of morbidity and almost always require inpatient care. However, these results require external verification in various population groups, as the single-center design restricts generalizability. Further prospective randomized controlled trials are needed to better define the efficacy and limitations of these indices.

## Figures and Tables

**Table 1:** Comparison of clinical features and inflammation indices calculated from hemograms collected at or before 5 weeks of gestation.

	NVP Group (*n* = 278)	Control Group (*n* = 278)	*p-*Value
Maternal age (years)	26 (22–29.2)	27 (23–31)	0.06
Gestational age (weeks)	9 (7–12)	9 (8–10)	0.333
BMI (kg/m^2^)	25 (21–31)	28.5 (26–31)	**<0.001**
Gravida (number)	1 (1–2)	2 (1–3)	**<0.001**
Parity (number)	0 (0–1)	1 (0–2)	**<0.001**
SII (10^3^/μL)	1173 (771–1702)	774 (599–1089)	**<0.001**
SIRI (10^3^/μL)	1.9 (1.3–2.7)	1.8 (1.4–2.5)	0.74
PIV (10^6^/μL^2^)	487.1 (321.7–816.8)	397.7 (283.9–663.4)	**<0.001**

Data are expressed as median (quartile 1–quartile 3). A *p* value of <0.05 indicates a significant difference. Statistically significant *p*-values are in bold. BMI: body-mass index; HG: hyperemesis gravidarum; NVP: nausea and vomiting in pregnancy; PIV: pan-immune inflammation value; SII: systemic immune-inflammatory index; SIRI: systemic inflammatory response index.

**Table 2:** Comparison of clinical features and inflammation indices calculated from hemograms collected at or before 5 weeks of gestation between different subgroups of NVP.

	Mild NVP (*n* = 58)	Moderate NVP (*n* = 140)	Severe NVP (HG) (*n* = 80)	*p-*Value
Age (years)	27 (22–30)	26 (22–29)	26 (23–30)	0.68
Gestational age (weeks)	9 (7–12)	9 (7–12)	10 (8–12)	0.454
BMI (kg/m^2^)	27 (22–31)	24 (21–31)	26 (23–31)	0.21
Gravida (number)	1 (1–2)	1 (1–2)	1 (1–2)	0.42
Parity (number)	0 (0–1)	0 (0–1)	0 (0–1)	0.41
SII (10^3^/μL)	808 (677–1397)	1209 (764–1703)	1234 (910–1792)	0.003 *
SIRI (10^3^/μL)	1.50 (1.00–2.21)	1.94 (1.29–2.76)	1.95 (1.48–3.38)	0.009 *
PIV (10^6^/μL^2^)	376 (264–582)	513 (347–819)	516 (368–950)	0.007 *

Data are expressed as median (quartile 1–quartile 3). A *p* value of <0.05 indicates a significant difference. Statistically significant *p*-values are in bold. *: significant differences between Mild NVP vs. Moderate NVP and Mild NVP vs. Severe NVP (HG) groups. BMI: body-mass index; HG: hyperemesis gravidarum; NVP: nausea and vomiting in pregnancy; PIV: pan-immune inflammation value; SII: systemic immune-inflammatory index; SIRI: systemic inflammatory response index.

**Table 3:** ROC curve analysis for hemogram-derived indices that can be used to predict NVP and the need for inpatient treatment.

	Variable	AUC	CI 95%	*p*	Cut-Off Value	Sensitivity (%)	Specificity (%)	+ LHR	− LHR
NVP prediction	SII (10^3^/μL)	0.708	0.67–0.75	**<0.001**	>966	63.67	68.35	2.01	0.53
	PIV (10^6^/μL^2^)	0.593	0.55–0.64	**<0.001**	>348	73.02	41.37	1.25	0.65
Inpatient treatment prediction	SII (10^3^/μL)	0.639	0.58–0.70	**0.002**	>1056	64.55	68.97	2.08	0.51
	SIRI (10^3^/μL)	0.625	0.57–0.68	**0.003**	32.12	77.27	43.10	1.36	0.53
	PIV (10^6^/μL^2^)	0.627	0.57–0.68	**0.003**	>350.19	77.27	46.55	1.45	0.49

A *p* value of <0.05 indicates a significant difference. Statistically significant *p*-values are in bold. AUC: area under the curve; CI: confidence interval; LHR: likelihood ratio; NVP: nausea and vomiting in pregnancy; PIV: pan-immune inflammation value; ROC: receiver operating characteristic; SII: systemic immune-inflammatory index; SIRI: systemic inflammation response index.

**Table 4:** Comparison of hemogram-derived indices that can be used to predict NVP and the need for inpatient treatment.

Variable	NVP Prediction		Inpatient Treatment Prediction		
	SII (AUC = 0.708)	PIV (AUC = 0.593)	SII (AUC = 0.639)	SIRI (AUC = 0.625)	PIV (AUC = 0.627)
SII		**<0.001**		0.661	0.698
SIRI			0.661		0.872
PIV	**<0.001**		0.698	0.872	

A *p* value of <0.05 indicates a significant difference. Statistically significant *p*-values are in bold. AUC: area under the curve; NVP: nausea and vomiting in pregnancy; PIV: pan-immune inflammation value; SII: systemic immune-inflammatory index; SIRI: systemic inflammation response index.

**Table 5:** Univariate and multivariate logistic regression analyses of inflammatory indices in predicting NVP.

	OR	95% CI	*p*
SII (10^5^/μL)	**1.187**	**1.138–1.239**	**<0.001**
SII (10^5^/μL) *	1.163	1.114–1.215	**<0.001**
SII (10^5^/μL) **	1.163	1.115–1.214	**<0.001**
PIV (10^8^/μL^2^)	1.103	1.052–1.157	**<0.001**
PIV (10^8^/μL^2^) *	1.085	1.034–1.139	**0.001**
PIV (10^8^/μL^2^) **	1.086	1.034–1.140	**0.001**

A *p* value of <0.05 indicates a significant difference. Statistically significant *p*-values are in bold. *: adjusted for BMI and gravida. **: adjusted for BMI and parity. BMI: body-mass index; CI: confidence interval; NVP: nausea and vomiting in pregnancy; OR: Odds ratio; PIV: pan-immune inflammation value; SII: systemic immune-inflammatory index.

## Data Availability

Within the scope of personal data protection, data related to the study will be made available only to the individual who requests it, and only after approval by the ethics committee of the institution where the study is conducted.

## Abbreviations

The following abbreviations are used in this manuscript:

AUC	Area under the curve
BMI	Body-mass index
CBC	Complete blood count
CI	Confidence interval
CRL	Crown-rump length
HG	Hyperemesis gravidarum
LHR	Likelihood ratio
MLR	Monocyte-to-lymphocyte ratio
NLR	Neutrophil-to-lymphocyte ratio
NVP	Nausea and vomiting in pregnancy
PIV	Pan-immune inflammation value
PLR	Platelet-to-lymphocyte ratio
PUQE	Pregnancy-unique quantification of emesis and nausea
ROC	Receiver operating characteristics
SII	Systemic immune-inflammation index
SIRI	Systemic inflammatory response index
